# Cloud-computing and machine learning in support of country-level land cover and ecosystem extent mapping in Liberia and Gabon

**DOI:** 10.1371/journal.pone.0227438

**Published:** 2020-01-10

**Authors:** Celio de Sousa, Lola Fatoyinbo, Christopher Neigh, Farrel Boucka, Vanessa Angoue, Trond Larsen

**Affiliations:** 1 Universities Space Research Association/GESTAR, Columbia, Maryland, United States of America; 2 Earth Sciences Division, NASA Goddard Space Flight Center, Greenbelt, Maryland, United States of America; 3 Agence Gabonaise d'Etudes et d'Observations Spatiales (AGEOS), Libreville, Gabon; 4 Conservation International, Arlington, Virginia, United States of America; University of the Aegean School of Social Sciences, GREECE

## Abstract

Liberia and Gabon joined the Gaborone Declaration for Sustainability in Africa (GDSA), established in 2012, with the goal of incorporating the value of nature into national decision making by estimating the multiple services obtained from ecosystems using the natural capital accounting framework. In this study, we produced 30-m resolution 10 classes land cover maps for the 2015 epoch for Liberia and Gabon using the Google Earth Engine (GEE) cloud platform to support the ongoing natural capital accounting efforts in these nations. We propose an integrated method of pixel-based classification using Landsat 8 data, the Random Forest (RF) classifier and ancillary data to produce high quality land cover products to fit a broad range of applications, including natural capital accounting. Our approach focuses on a pre-classification filtering (Masking Phase) based on spectral signature and ancillary data to reduce the number of pixels prone to be misclassified; therefore, increasing the quality of the final product. The proposed approach yields an overall accuracy of 83% and 81% for Liberia and Gabon, respectively, outperforming prior land cover products for these countries in both thematic content and accuracy. Our approach, while relatively simple and highly replicable, was able to produce high quality land cover products to fill an observational gap in up to date land cover data at national scale for Liberia and Gabon.

## Introduction

Degradation from illegal logging, shifting cultivation and industrial and commodity crops pose some of the greatest threats to tropical forests [1,2]. For instance, from 2000 to 2011, 40% of tropical deforestation came from commodity crop production [3]. Although there has been an increasing number of agribusiness companies making zero-deforestation pledges, the halt of deforestation worldwide is far from being achieved. Sub-Saharan Africa has shown one of the fastest rates of cropland expansion [4] and highest deforestation rates [5]. The growing demand for land and more stringent environmental and land use regulations in Southeast Asia and South America may be incentivizing the transition of export-oriented commodity crop expansion to Sub-Saharan Africa, where agriculturally suitable land and labor are abundant and cheap. This has increased the pressure on forests, biodiversity, natural resources and ecosystems in this region. The Guinean Forest region in West Africa stretches from Guinea to west of Cameroon and it is separated by the Dahomey gap into the Upper and Lower Guinean Forests. Almost half of what remains of the Upper Guinean Forests is found in Liberia, a biodiversity hotspot and it is one of the highest global conservation priorities [6]. Further south, in the Congo-Ogoué basin region of Central Africa, Gabon is the second most forested tropical country in the world with 88.5% forest cover and 23.5 M ha of forest [7]. While Central African forests still have much lower deforestation rates of 0.1% per year [5], Gabon’s significant forest extent that is relatively well-preserved makes its conservation and management all the more crucial [7].

In recent years, an increasing amount of research has been devoted to understanding the importance of these ecosystems and their wide range of services that are important for human well-being [8–11]. Liberia together with nine other African countries is a member of the Gaborone Declaration, pledging to "integrate the value of nature into their national policies and programs, recognizing that nature is needed for economic growth and sustainability". Over time, the use of ecosystem services beyond sustainable supply levels will lead to ecosystem depletion and/or degradation. In this context, estimating the multiple services obtained from ecosystems is vital to support decision-making processes and to avoid exploitation that fails to promote sustainable growth. However, to date, input data needed to carry out rigorous natural capital accounting have been lacking.

The initial step in an ecosystem accounting framework is monitoring and quantifying the temporal and spatial dynamics of land cover and/or ecosystems. The remote sensing community has widely relied on existing global land cover datasets and these products have played an important role in the estimates of land cover across the globe. However, along with the different land cover definitions, differences in land cover extent estimates may also arise from differences in data sources, methods and coarse spatial resolution of these products. For instance, there is a Moderate-resolution Imaging Spectroradiometer (MODIS) Collection 5 Land Cover Product at 500 m spatial resolution [12] while the European Space Agency’s GlobCover product is based on ENVISAT-MERIS data and it has a 300 m spatial resolution [13,14]. Other examples include: The SPOT4-based Global Land Cover (GLC2000) map with a 1 km resolution (lower at higher latitudes) [15,16] and the Global Land Cover Characterization database (GLCC) based on the Advanced Very High Resolution Radiometer (AVHRR) with 1 km resolution [17].

While there have been efforts to produce nationwide land cover databases for both developed (e.g. [18]) and developing (e.g.[19]) countries, currently, consistent, country-wide land cover maps based on Liberia’s recently defined forest definition (i.e 30% minimum canopy cover, a 5-meter minimum height and a 1ha minimum area) do not exist, while Gabon has yet to adopt a national definition of forest. Most studies focused either on certain portions of the country, at coarse resolution or using arbitrary forest definitions [20–23]. Even though existing mapping approaches yield valuable results, there is potential for a more comprehensive and systematic utilization of multi-sensor, multi-temporal earth observation data. Until very recently, the high computational power and the difficulties to distribute non-trivial classification algorithms across multiple computational workstations has challenged classification approaches aimed at producing accurate high-resolution land cover maps on country and global scales. As a response to this challenge, Google^TM^ developed a geospatial data analysis platform–Google Earth Engine (GEE)–capable of storing and analyzing vast amounts of remote sensing data [24]. GEE's computing infrastructure revolutionizes time-consuming remote sensing processes, facilitates access to a large catalogue of Earth observation data, and paves a new way forward for rapid land cover classification.

The availability of large-volume remote sensing data through GEE and its advanced machine learning tools, namely the Random Forest (RF), have been already leveraged for mapping the land cover dynamics worldwide. Random Forest is a well-known classification method and its robustness (compared to other well established methods such as Decision Trees and Support Vector Machines) has been proven and confirmed over the years, as seen in the literature (e.g. [25–29]). Similarly, the Landsat archive available on GEE has been used to develop widely used global dataset at 30-m spatial resolution such as the global forest cover [30], global mangrove extent [31] and most recently, cropland extent for southeast and northeast Asia [32]. The studies that have focused in one or more components of GEE (e.g. as a data source or as a processing platform) mostly focused on provincial- and regional-scales [33–35], coarse spatial resolution [36] or on a single land cover type [29,37–41]. GEE offers an opportunity for developing moderate to high resolution country-level land cover datasets based on country-specific needs and land cover types. However, in order to produce reliable and accurate wall-to-wall land cover maps, new systematic classification methods and approaches need to be developed to deal with the complex classification issues, such as cloud coverage, poor image quality and high data volumes.

In our study, the Landsat 8 Operational Land Imager Surface Reflectance imagery archive available on GEE was used to produce a 30-m resolution land cover and impervious surface map for Liberia and Gabon circa 2015. We propose an integrated method of pixel-based classification using Random Forest (RF) classifier and ancillary data to develop a land cover map product that satisfy the requirements of a broad range of applications, including natural capital accounting. The intent of this study was to demonstrate how cloud-based frameworks such as GEE can help policy-makers and scientists in developing nations, where high-performance computational resources may be lacking, to generate accurate land cover maps that can be used in different applications. Moreover, our intent was to leverage GEE’s seemingly straightforward technology transferability to deliver a designed approach and empower these nations to maintain the ongoing natural capital accounting efforts to monitor change over time.

## Materials and methods

**Fig 1:**
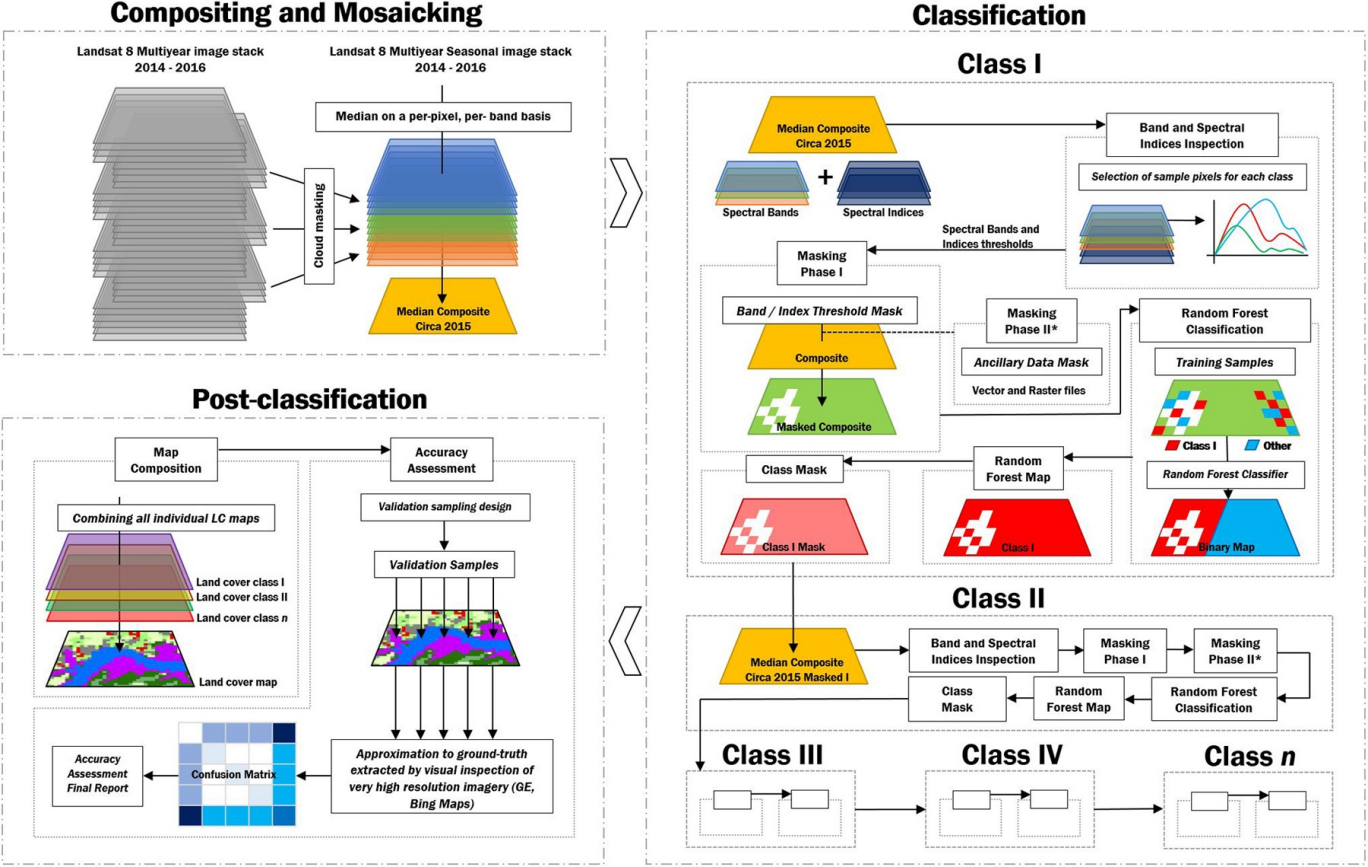
Schematic diagram of the methodology. Processes and respective sub-processes are represented by the different dashed boxes. Each dashed box is explained in the following sections.

A comprehensive overview of the methodology is shown in Fig 1. The preprocessing, classification methodology and post classification steps which were applied in the mapping approach for Liberia and Gabon are presented in the following sub-sections. The code repository used for the mosaicking and classification process is available here: https://github.com/celiohelder/Liberia-Classification

### Input data and imagery composition

In this study, the Landsat 8 Operational Land Imager Surface Reflectance imagery archive available on GEE was used. GEE’s cloud screening algorithm based on quality assessment bands (QA) was applied in order to remove cloud and cloud shadow contaminated pixels for each of the Landsat scene covering Liberia and Gabon. Creating an annual 30-m cloud-free imagery composition for Liberia is a challenging task because of the west African monsoon [42], which causes constant clouds across the Gulf of Guinea most of the time. The rainy season in Liberia ranges from May to October and it frequently rains in other months, except in the short dry season that runs from December to February/March. In Gabon, 80% of precipitation occurs during the two rainy seasons ranging from March to May and October to November. Thus, multi-year seasonal composites were necessary in attaining wall-to-wall cloud-free mosaics over Liberia and Gabon using Landsat 8 (S1 and S2 Figs). For Liberia, a 2015 dry seasonal (December 2015 –March 2016) composite was produced by calculating the median values from images from the central year (2015), plus and minus one dry season: December/2014 and March/2015 (Dry Season 2015), December/2015 and March/2016 (Dry Season 2015) and December/2016 and March/2017 (Dry Season 2016). For Gabon, the 2015 annual composite was produced by calculating the median of all cloud-masked pixels from the images available for the central year (2015), plus and minus one year (2014 and 2016). We reduced the seasonal (Liberia) and annual (Gabon) image collection into a median composite using GEE’s per-band, per- pixel approach. A median composite on a per-band, per-pixel approach is currently state of the art in GEE and was also used in several studies worldwide mapping land cover extent and land cover change from regional to continental scales [27,29,32,36,43,44]. A multi-year compositing approach was adopted to ensure that cloud-free pixels were included in the 2015 composites. A similar approach using multi-year composite was also used in [44].

**Fig 2:**
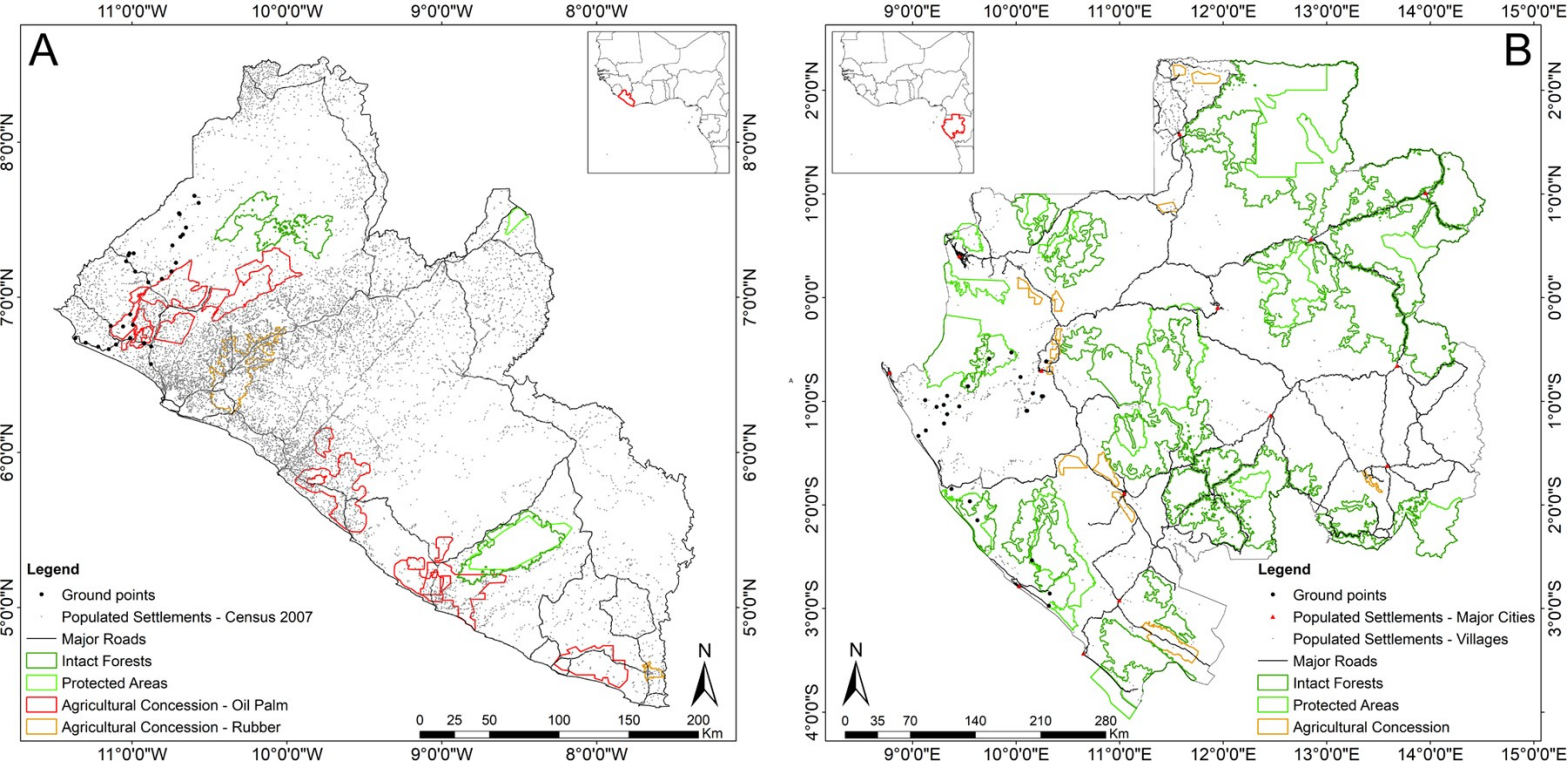
Geospatial datasets used as support in mapping and validation for Liberia and Gabon. Full list of datasets and their sources and references are provided in the text and Table 2.

In addition to the Landsat 8 imagery, several additional geospatial datasets were used as support in mapping and validation (Table 1 and Fig 2).

**Table 1:** List of ancillary data.

Name	Description	Source
Photos	150 photographs for 33 selected locations in Liberia	Trond Larsen, Conservation International
Administrative borders	Country borders of Liberia and Gabon	GADM ^a^ and PNAT Gabon ^b^
Concessions boundaries and other areas	Agricultural (Palm Oil and Rubber) and Mining concessions, intact forest and protected areas	LIGIS ^c^ and PNAT Gabon ^b^
Settlements	Location of every populated settlement in Liberia as of Census 2007/2008	LIGIS ^c^ and PNAT Gabon ^b^
Infrastructure	Lines of streets and roads	AICD ^d^ and PNAT Gabon ^b^
Land cover	Existing land cover data (Giri 2010, GlobCOVER 2009, CCI ^1^ ESA Africa Land Cover 2016, Gabon Forest Cover)	[45], [13,14] and AGEOS ^e^
Forest Cover	Global Forest extent and change [30]	Global Forest Change Landsat Forest Cover Change

^1^ Climate Change Initiative–European Space Agency.

^a^ Global Administrative Boundaries https://gadm.org/.

^b^ Plan National d'Affectation des Terres (PNAT) ^b^ http://pnatgabon.ga/.

^c^ Liberia Institute of Statistics and Geo-information Services http://www.lisgis.net/.

^d^ Africa Infrastructure Country Diagnostic.

^e^ Agence Gabonaise d'Etudes et d'Observations Spatiales.

### Random forest and training data

In this study, the RF classification method was used. The RF classification algorithm is described in detail in [46]. Briefly, the RF classifier is an ensemble of classification trees, where each tree contributes with a single vote for the assignment of the most frequent class to the input data. Different from Decision Trees, which use the best predictive variables at the split, RF uses a random subset of the predictive variables in order to reduce the generalization error. The RF classifier within GEE was used to model the land cover classes for Liberia and Gabon.

Training data was acquired by visually inspecting freely available high spatial resolution imagery (e.g. Google Earth). The same approach was also adopted by [44,47–49]. In this study, we focused on imagery from the 2015–2018 period and selected the corresponding Landsat pixels to serve as training data for 10 land cover classes (Table 2). Instead of single pixels, each sample consisted of a polygon containing a variable number of relatively homogenous pixels of a given land cover class. These pixels were then used as training inputs for the RF classifier (Table 2). The number of samples varied depending on the land cover class and its availability of homogenous areas. Training samples were selected across Liberia and Gabon with the aim of capturing more than 500 sample pixels for each class in each country to ensure that the training data contained samples that were representative of each land cover class. Moreover, we used a total of 33 ground truth points for artificial surfaces, grasslands, woody crops, and the three tree covered area classes for Liberia and 26 ground truth points for flooded forests in Gabon as an additional set of training data (See Fig 2). At each location in Liberia, 4 photographs were taken following the cardinal directions. An example of these photographs is shown in the S3 Fig.

**Table 2:** Class definitions and training data for the Liberia and Gabon land cover maps.

Class level 1	Description	Training sample size
Water bodies ^a^^,^ ^b^	All areas of open water	5529 ^a^, 38840 ^b^
Mangroves and Wetlands ^a^^,^ ^b^	Areas where trees ^2^ account for more the majority of vegetative cover and the soil or substrate is saturated with or covered with water.	8079 ^a^, 5763 ^b^
Artificial Surfaces (Human Settlements) ^a^^,^ ^b^	Buildings and other man-made structures cover 50–100% of the surface.	4062 ^a^, 6154 ^b^
Barren Land ^a^^,^ ^b^	Areas characterized by bare rock and/or exposed soil with little or no “green” vegetation present.	8771 ^a^, 3608 ^b^
Ecosystem Complex (Sand and Intertidal areas) ^a^	Areas of sand beaches and areas that are submerged during high tide and exposed during low tide.	506 ^a^
Woody Crops (Rubber and Palm) ^a^^,^ ^b^	Areas dominated ^1^ by non-natural woody vegetation. Usually followed by harvest and bare soil and/or grassland/mixed vegetation period.	30549 ^a^, 17548 ^b^
Grasslands ^a^^,^ ^b^	Areas dominated ^1^ by herbaceous types of cover.	19458 ^a^, 11393 ^b^
Tree Covered Areas (Dense/Primary) ^a^^,^ ^b^	Areas dominated ^1^ by broadleaved trees ^2^ relatively intact or no clearly visible indication of human activity.	2122 ^a^, 5689 ^b^
Tree Covered Areas (Open/Secondary) ^a^^,^ ^b^	Areas dominated ^1^ by broadleaved trees ^2^ with evidence of human disturbance, logging history, >15–20 years from regeneration if cleared.	516 ^a^, 965 ^b^
Mixed Vegetation ^a^	Areas dominated ^1^ by broadleaved trees ^2^, shrubs, bushes and herbaceous vegetation.	2087 ^a^,
Flooded Forests ^b^	Tree-dominated areas along rivers and streams subject to dramatic water fluctuations and seasonal flooding.	-

^a^ Land cover classes included in the Liberia Land Cover Map and training sample size.

^b^ Land cover classes included in the Gabon Land Cover Map and training sample size.

^1^ Presence equal or greater than 50% within a 30-meter pixel (900 m^2^).

^2^ Woody vegetation with height equal or greater than 5 meters.

### Modeling and classification

Following the cloud masking and mosaicking/compositing phase, the land cover classification was performed. We adopted a binary classification (Class/Non-Class) strategy where each land cover class was mapped individually. For a given land cover class, all 9 other land cover classes were collapsed and treated as a single class “Other”. For a given land cover class, the following steps were taken:

- Pixel-based classification may generate a large number of misclassified pixels (the ‘‘salt-and-pepper effect”) due to the spectral diversity within the same land cover type and spectral confusion between land cover types. Thus, we included a pre-classification masking phase: A set of pixels were sampled for each class and its average reflectance values for Landsat spectral bands and spectral indices (Table 3) was computed. A spectral signature analysis was performed for identifying potential spectral bands and reflectance-based spectral indices thresholds for differentiating between two classes: the land cover class being mapped and the remaining 9 land cover classes merged as a single class called “Other”.
- The threshold values were then used to mask pixels in the Landsat composite in order to isolate only pixels of the land cover class being mapped (Fig 1 –Masking Phase I). Tables 4 and 5 show the set of spectral and spatial thresholds used to mask the majority of pixels not belonging to a given class. With respect to minimizing the likelihood of introducing omission error while masking, the threshold values were selected in order to include all the pixels of a given class plus some extent of other classes.

**Table 3:** Reflectance-based spectral indices used as predictors for masking phase and classification.

Category	Name		Formulation^1^	Reference
Water Bodies and Mangroves/Wetlands	NDMI	Normalized Difference Mangrove Index	(B_7_ – B_3_)/(B_7_ + B_3_)	[50]
	MNDWI	Modified Normalized Difference Water Index	(B_3_ – B_6_)/(B_3_ + B_6_)	[51]
Barren Land and Artificial Surfaces	NDBI	Normalized Difference Built-up Index	(B_6_ – B_5_)/(B_6_ + B_5_)	[52]
	NDII	Normalized Difference Impervious Index	(B_3_ – B_10_)/(B_3_ + B_10_)	[53]
	NDBaI	Normalized Difference Bareness Index	(B_6_ – B_10_)/(B_6_ + B_10_)	[54]
	BI	Bare Soil Index	(B_6_ – B_7_)/(B_6_ + B_7_)	[55]
	UI	Urban Index	(B_7_ – B_5_)/(B_7_ + B_5_)	[56]
Green Vegetation	NDVI	Normalized Difference Vegetation Index	(B_5_ – B_4_)/(B_5_ + B_4_)	[57]
	GCVI	Green Chlorophyll Vegetation Index	(B_5_/ B_3_) - 1	[58]
	SR	Simple Ratio	B_5_/ B_4_	[59]
Band Ratios	R75	B_7_B_5_ Ratio	B_7_/ B_5_	-
	R65	B_6_B_5_ Ratio	B_6_/ B_5_	-
	R34	B_3_B_4_ Ratio	B_3_/ B_4_	-

^1^ B_*i*_ represents Landsat 8 OLI band that matches the original formulation

**Table 4:** Spectral and spatial thresholds for the pre-classification masking phase I and II for Liberia.

Class	Spectral and Spatial Thresholds
Water bodies	MNDWI > - 0.2
Mangroves	MNDWI > - 0.35
	NDVI > 0.3
Artificial Surfaces	B6 > 0.25
	NDVI < 0.45
	Location of every human settlement as of census 2007 1
Ecosystem Complex	R65 > 0.8
	1000 m buffer from shore
Barren Land	B6 > 0.25
	NDVI < 0.45
Grasslands	R65 > 0.8
	NDVI < 0.4
Woody Crops	R65 > 0.6
	Agriculture Concessions provided by LISGIS 1
Tree Covered Areas (Dense/ Primary)	B6 > 0.14
Tree Covered Areas (Open/ Secondary)	B5 > 0.28

^1^ Ancillary data used in the Masking Phase I

**Table 5:** Spectral and spatial thresholds for the pre-classification masking phase I and II for Gabon.

Class	Spectral andSpatial Thresholds
Water bodies	MNDWI > - 0.23
Mangroves	SRTM < 95
	MNDWI > - 0.3
	NDVI > 0.35
Artificial Surfaces	UI > - 0.568
	Location of every human settlement provided for 2013 (PNAT)^1^
Barren Land	UI > - 0.568
	After masking artificial surfaces to avoid confusions
Grasslands	R65 > 0.59
	NDVI < 0.45
Woody Crops	R65 > 0.6
	Agriculture Concessions provided by PNAT ^1^
Tree Covered Areas (Dense/Primary)	Gabon Forest Cover Extent 2015 provided by AGEOS

^1^ Ancillary data used in the Masking Phase II

Classes such as Mangroves and Grasslands required a secondary spectral threshold for both Liberia and Gabon composites. Artificial Surfaces, Barren Lands and Grasslands also required a secondary threshold in order to be accurately isolated in Liberia. These classes are likely to be very heterogeneous and often show intermixing with other land cover classes. Moreover, for classes with high spectral similarity, additional masks were created based on ancillary datasets and nature-based knowledge (Fig 1 –Masking Phase II). For instance, to avoid confusion between artificial surfaces and barren land pixels, we constrained the classification of artificial surfaces pixels within a buffer area based on each human settlement location. This technique reduced the number of pixels that was ultimately grouped into the “Other” class. Hence, decreasing the processing time and avoiding memory limit errors on GEE. Moreover, this pre-classification filtering phase assures that a significantly smaller number of pixels is prone to be misclassified by the RF classifier, likely decreasing errors of omission and commission of the final land cover maps.

- Pixels from the resulting masked composites were then classified using a RF classifier within GEE and the training samples. In our study, the user-defined parameter ‘number of trees’ was set to 100 and the Landsat spectral bands and spectral indices described in Table 3 were used as predicting variables in the RF model depending on the category of each land cover class. The final classification was a binary output of Class_*i*_ and Other (Fig 1 –Random Forest Classification).
- A knowledge-based verification was then carried out to check and improve the resulting classification outputs. For instance, water and mangroves/wetlands occur in relatively flat or low relief areas. With such knowledge, slope could be calculated from the Shuttle Radar Topography Mission (SRTM) data and used to identify errors of omission or commission and as another predictor for the Random Forest classifier. Another typical example is sand and intertidal areas classes in Liberia have a specific geographical distribution along the shore line. This knowledge is indispensable to identify errors and confusion with other spectrally similar classes (e.g barren land).
- The other land cover classes (“Other”) was excluded from the classification output and the land cover class being mapped (Class_*i*_) was stored and further converted into a mask to be used in the Landsat 8 composite prior the next land cover class classification (Fig 1 –Class Mask). This process was repeated until all classes were mapped (Fig 1 –Classification).
- The final land cover maps for Liberia and Gabon were composited by combining individual land cover classes into a single digital raster map. The map was color coded and exported as a GIS-compatible GeoTIFF format.

### Flooded forests classification in Gabon

**Fig 3:**
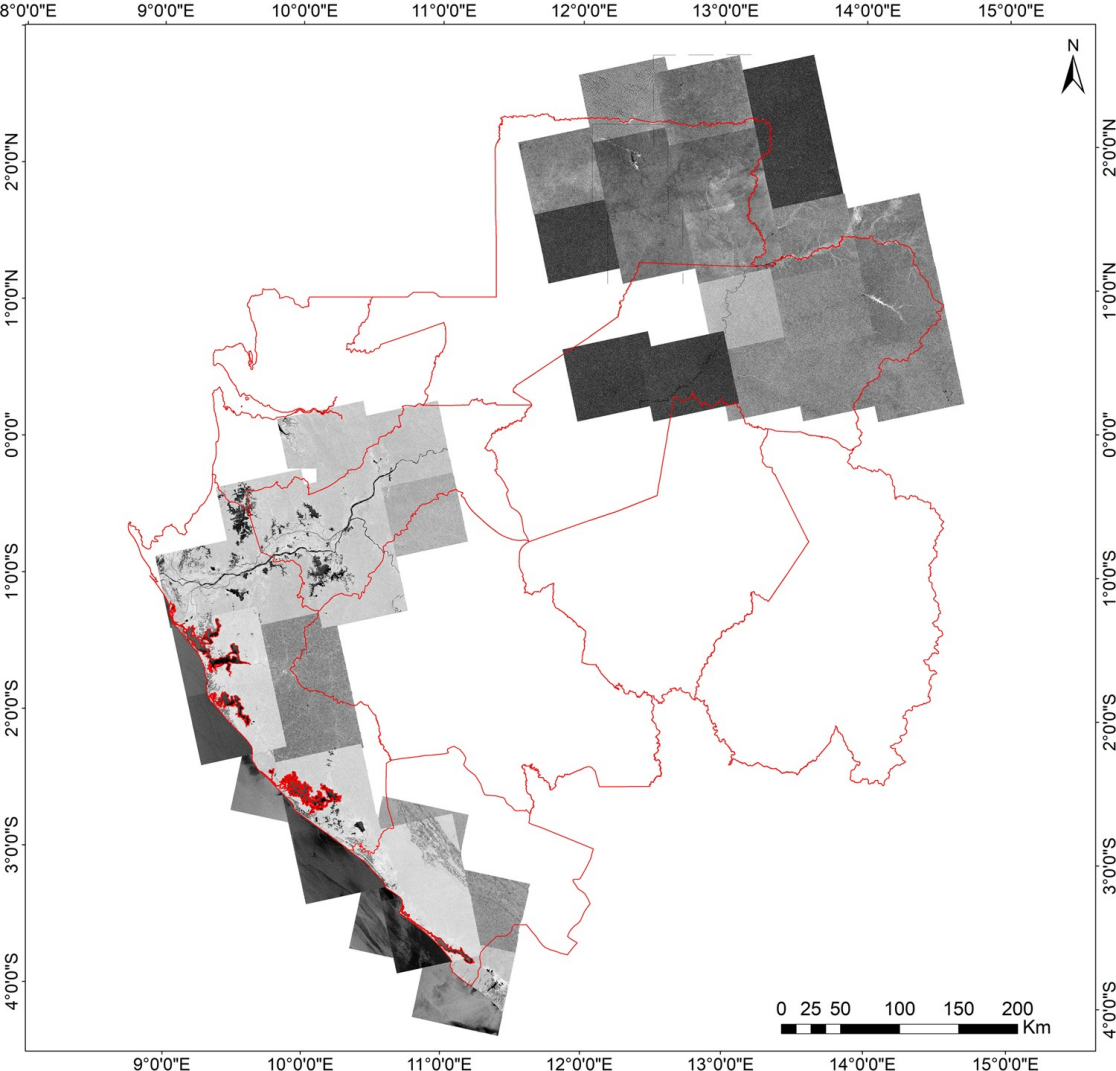
Advanced Land Observing Satellite-1 (ALOS) PALSAR's L-band SAR imagery over northeast and south-southwest provinces. Available images over Gabon for the dry and rainy seasons of the years of 2008 and 2009 were used.

Flooded forests provide valuable ecosystems services for both humans and wildlife and are found from south-west to north-east of Gabon. Although present in the Middle Ogooue and Ogooue Maritime provinces (see Fig 3B), the on-ground efforts to locate and map the flooded forests areas in Gabon have been limited by the dynamic nature and, most importantly, the inaccessibility of these areas (namely Woleu-Ntem and Ogooue-Ivindo provinces). We took advantage of synthetic aperture radar (SAR) imagery in Gabon for two primary reasons: 1) in highly cloud-covered areas such as Gabon, imaging radar is the only sensor that can provide consistent, periodic data in a reliable manner; and 2) optical systems (e.g. Landsat) are also limited by their inability to penetrate vegetation canopies whereas radar systems can, to some degree, provide sub-canopy information. Several studies showed that L-band (wavelength ~23 cm) SAR sensors are very suited to detect water under forest canopies (e.g. [60–66]) whereas it may not be detectable at C-band (wavelength ~6 cm). Thus, the Advanced Land Observing Satellite-1 (ALOS) PALSAR's L-band SAR imagery was acquired for Gabon’s dry (October-November and February-May) and rainy season (July-September and December-January) for the years of 2008 and 2009 for mapping permanent and seasonally flooded forests in the southwest (Middle Ogooue and Ogooue Maritime provinces) and northeast (Woleu-Ntem and Ogooue-Ivindo provinces) of Gabon (Fig 3).

We considered forests permanently flooded those areas detected during both dry and rainy season images while seasonally flooded forests were represented by the areas detected only on the rainy season images. Images acquired with HH single polarization mode (horizontal receive and horizontal transmit) were used because modeling efforts by [64] confirm that the ratio of backscatter from the flooded forest to that from the non-flooded forest is higher at HH polarization. Thus, making it suitable for flooded forest mapping. Because flooded forests in Gabon usually occur in the low, flood-prone areas along rivers, they were detected using the amplitude values of the HH bands and a digital elevation model derived from SRTM data for each ALOS PALSAR scene was considered. The flooded forest classes produced with ALOS PALSAR’s L-band imagery were further converted into a mask and used in the Landsat 8 composite prior to the classification of the Tree-covered areas classes.

### Post-classification and accuracy assessment

**Fig 4:**
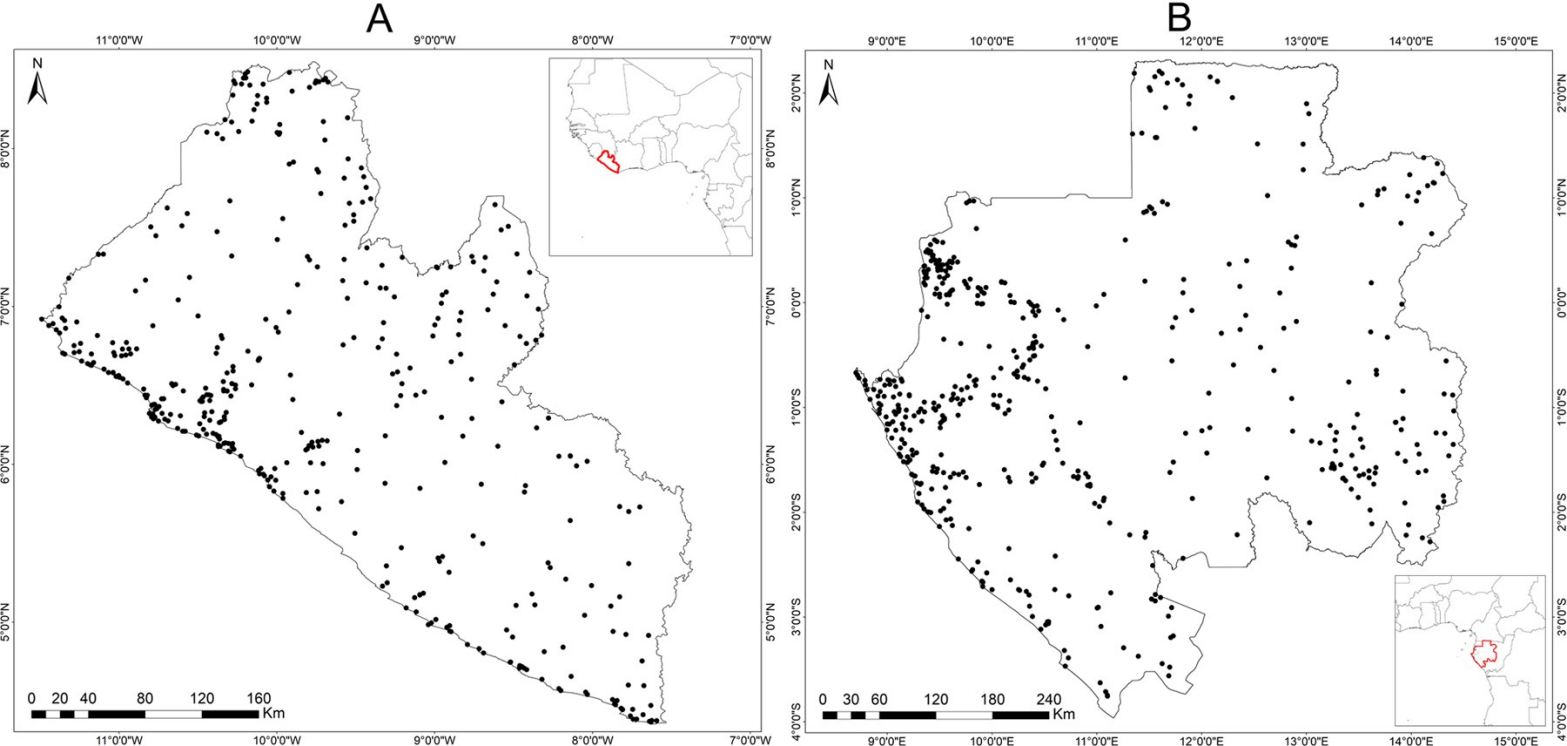
**Spatial distribution of the 500 validation samples (50 for each land cover class) for the Liberia (A) and Gabon (B) land cover map.** The locations where the 30 x 30 m footprint samples have been generated are shown as black points.

Validation of land cover maps based on rigorous sampling methods and high quality contemporaneous reference data is clearly desirable, however, as is very often the case, limited resources make fully rigorous quantitative validation an unreachable ideal. Nonetheless, we evaluated our land cover maps with other local datasets and performed qualitative validation. To attain equal representability of each class in the accuracy assessment, a stratified random pixel sampling design was used [67]. Similar weight was assigned to each class where a total of 500 samples (50 per each class) was generated (Fig 4). A sample with 30 m x 30 m footprint was used to match Landsat’s nominal spatial resolution to avoid unnecessary inflation of the overall accuracy of the final map.

A visual interpretation of very high resolution imagery available on Google Earth was carried and the corresponding dominant land cover class (more than 50% of the sample’s footprint) was assigned to each sample. Accuracies of the land cover product was carried out by comparing the reference samples derived from visual interpretation with the classification information contained in the map using an error matrix approach. Classification accuracies were assessed using overall accuracy, user’s and producer’s accuracy and Portmanteau Accuracy (binary class accuracy). In addition, a comparison was performed with other available land cover map of Liberia produced by [68] for the year 2014 to show spatial similarity. Although it does not have a comparable spatial resolution (10 m), this dataset was chosen because it is the latest land cover land use (LCLU) product available for Liberia. Finally, we also performed qualitative validation with the help of local experts in Monrovia, Liberia on September, 24^th^– 28^th^ 2018.

## Results

### RF output and accuracy assessment

**Fig 5:**
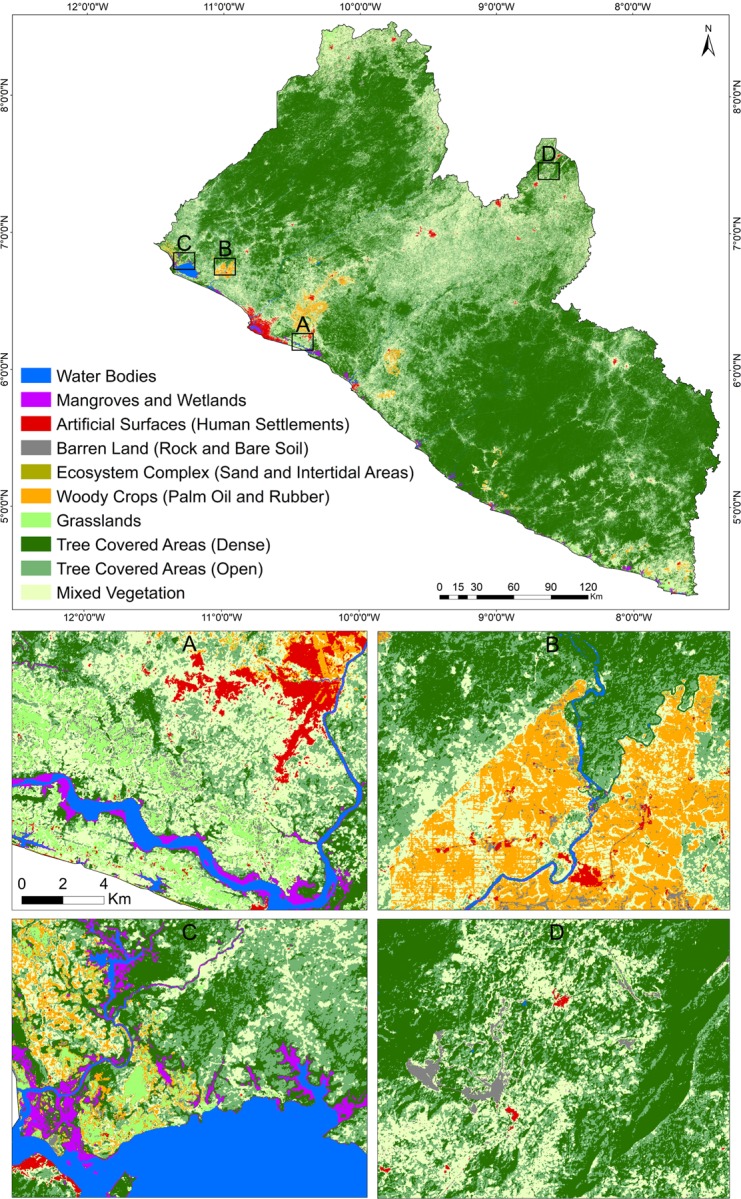
Random Forest model predicted land cover map for Liberia 2015. A-D insets are regional subsets showing different land cover classes across Liberia in finer detail.

**Fig 6:**
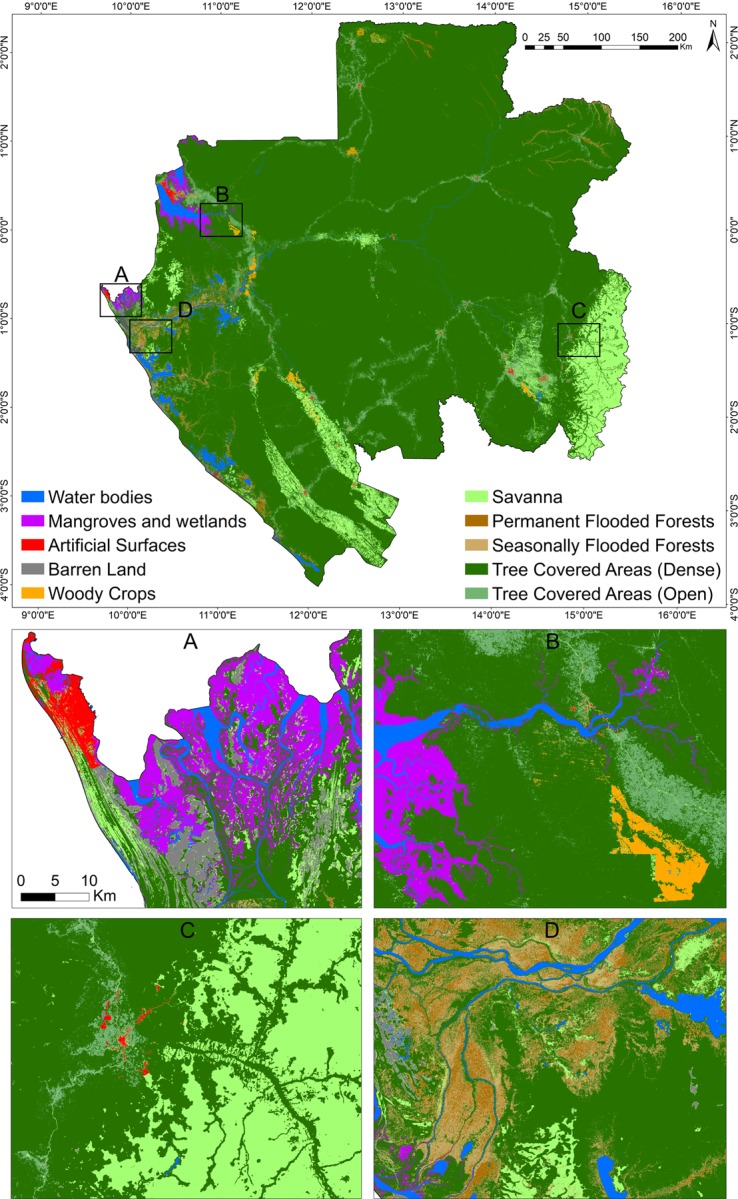
Random Forest model predicted land cover map for Gabon 2015. Regional subsets showing different land cover classes across Gabon in finer detail are shown (A-D).

The 2015 Liberia and Gabon land cover maps consisting of ten classes (Table 2) were generated based on our semi-automated methodology and the random forest classification model within GEE (Figs 5 and 6).

**Fig 7:**
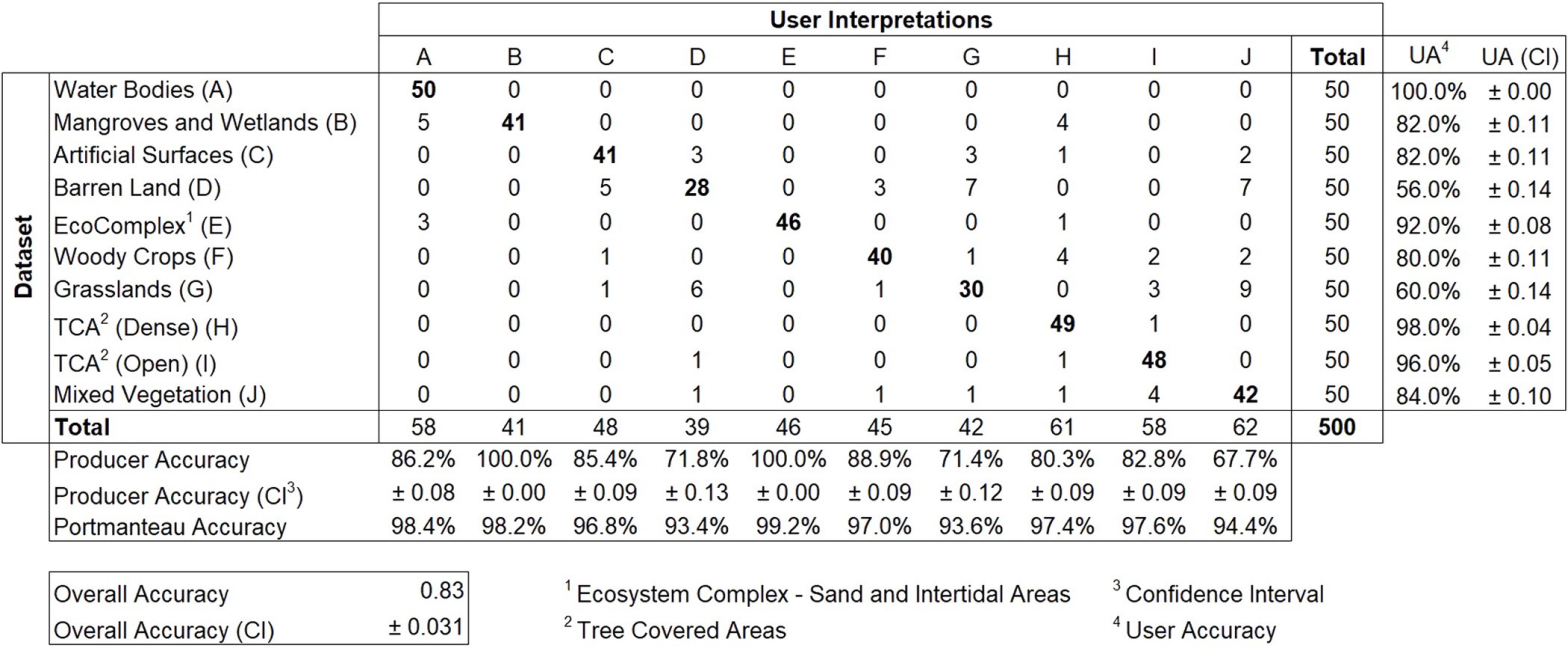
Error matrix for the Liberia land cover map.

**Fig 8:**
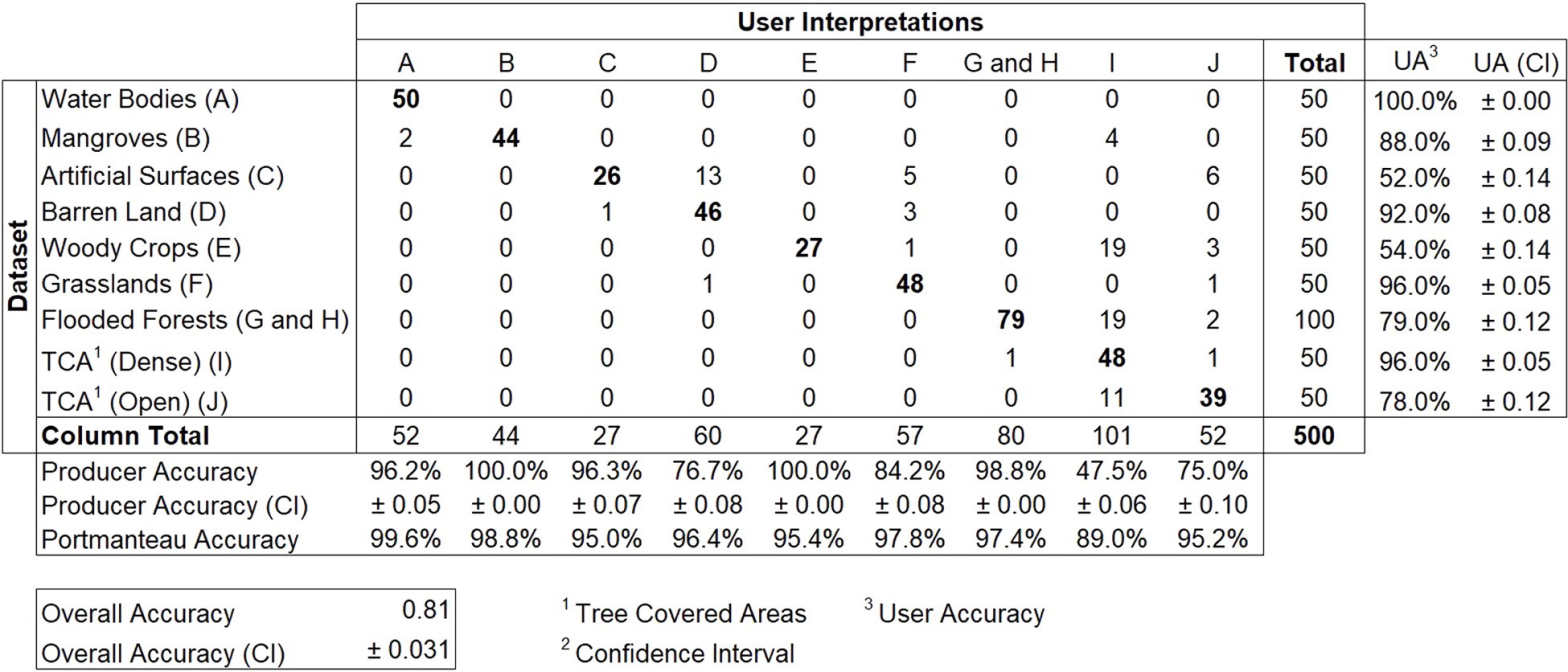
Error matrix for the Gabon land cover map.

The confusion matrices produced using the reference data acquired across the 10 classes in Liberia and Gabon are shown in Figs 7 and 8, respectively. Results were satisfactory, with an overall accuracy (percent correctly classified) of 83% for Liberia and 81% for Gabon. These overall accuracies can be considered as high as classes were not aggregated into the level 1 scheme of CORINE or into a landscape domain class for the accuracy assessment. For instance, the latest land cover product for Liberia (68) produced an overall accuracy of 91% when classes were collapsed into the three landscape domains for accuracy assessment. Of the 85 validation points assigned as misclassified in the Liberia map, 42 (49%) were due to misclassification of Barren Land and Grasslands into other classes while 13 were due to confusion between these two classes. Such areas are often characterized by a very heterogeneous, small-scale pattern of grasses intermixed with bare surfaces, resulting in confusion between the spectral signatures or sometimes an interpreter’s mislabeling. For Gabon, of the 93 validation points assigned as misclassified on the map, 47 (~50%) were due to misclassification of Artificial surfaces and Woody crops. Artificial surfaces were mostly misclassified as Barren Land (13 samples) while Woody crops were mostly misclassified as Tree Covered Areas (Primary) (19 samples). Human settlements in Gabon often showed large patches of exposed soil resulting in confusion between the spectral signature by the classifier. Similarly, mature woody crops’ spectral signature was very similar to the spectral signature of dense tree cover areas. Moreover, these classes were often next to each other increasing the chance of misclassification of edge pixels.

The land cover classes’ user’s and producers’ accuracy ranged from 56 to 100% and 68 to 100%, respectively, for Liberia. Gabon’s user’s accuracy ranged from 52 to 100% while its producer’s accuracy ranged from 47.5 to 100%. A 95% confidence interval for class specific user’s and producers’ accuracy and overall accuracy was also reported. For Liberia, water bodies were most accurately classified by the RF model with user’s and producer’s accuracy of 100 and 86%, respectively, followed by Mangroves with 82 and 100%, while Barren land was least accurately classified by the RF model with user’s and producer’s accuracy 56 and 72% respectively. Similarly, Gabon’s most accurately classified class was Water with user’s and producer’s accuracy of 100 and 86%, respectively, followed by grasslands with 96 and 84%. Although woody crops showed no error of omission (producer’s accuracy of 100%), it yielded an error of commission of 46% (user’s accuracy of 54%), along with artificial surfaces with a producer’s and user’s accuracy of 96.3 and 52%. For the accuracy assessment of seasonal and permanent flooded forests, we collapsed the two classes into a single class ‘Flooded Forests’ (G and H in the Error Matrix). This was necessary because: 1) no information about whether the ground truth points (Fig 2) were from seasonal or permanent flooded forests, and 2) from an optical high resolution imagery perspective, visually interpreting whether a given pixel sample is seasonal of permanent is still challenging due to the temporal density of cloud free observations. The flooded forest class yielded a user’s accuracy of 79% with the majority of its error of omission due to confusion with tree covered areas (primary).

The Binary Class Accuracy (Portmanteau Accuracy) describes the overall accuracy when the data are collapsed to two classes, the land cover type of interest, and all other land cover types combined into a single class. Ecosystem complex showed the highest binary class accuracy (99.2%) followed by water bodies (98.4%) and mangrove and wetlands (98.2%) in Liberia. For Gabon, water bodies yielded the highest binary class accuracy (99.6%) followed by mangroves (98.8%) and grasslands (97.8%).

### Flooded forests from ALOS PALSAR 1

**Fig 9:**
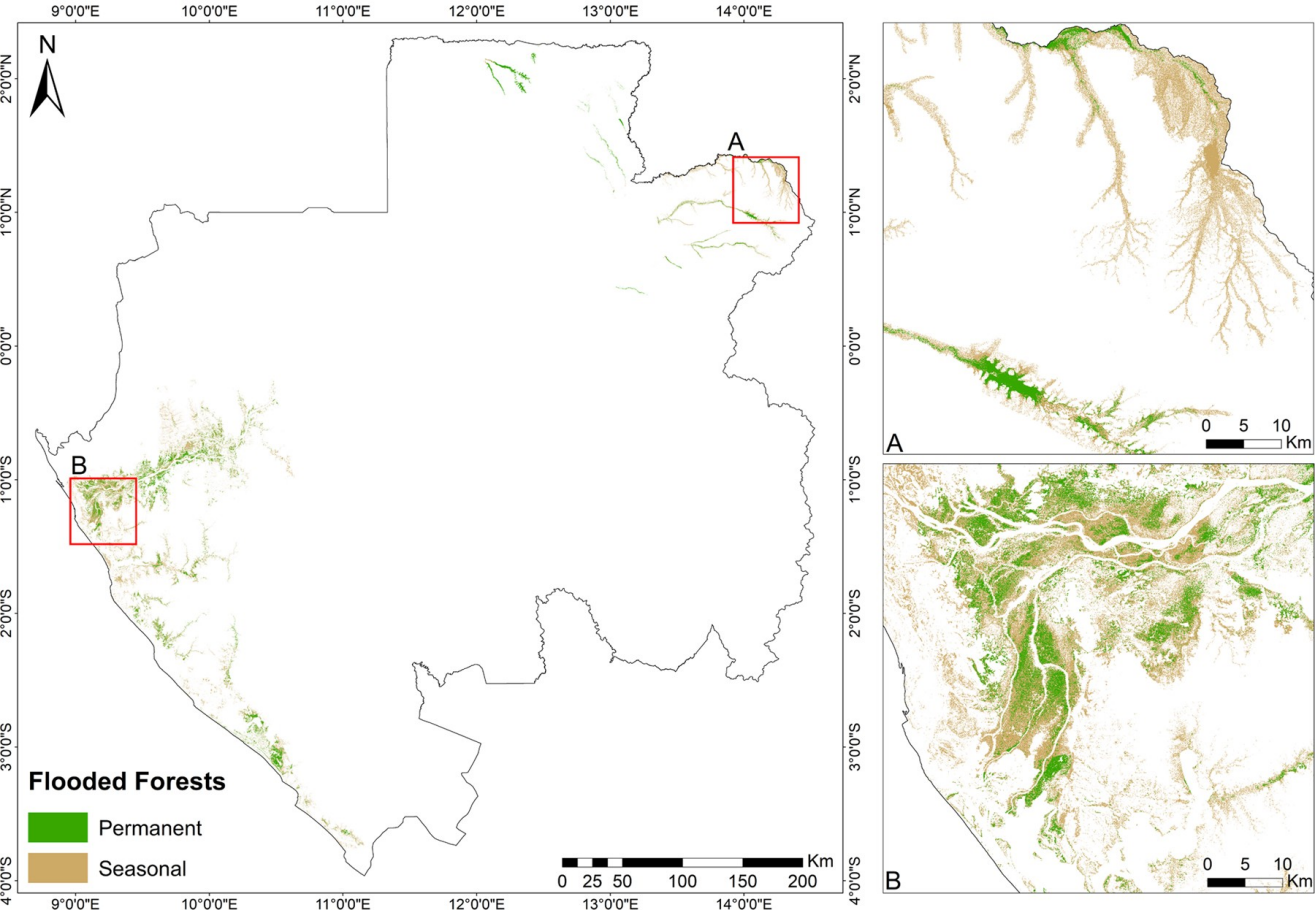
Seasonally and permanent flooded forests in Gabon derived from ALOS PALSAR's L-band SAR imagery. (A) and (B) show details of flooded forests in the Middle Ogooue and Ogooue-Ivindo provinces, respectively.

Fig 9 shows the spatial distribution of seasonally and permanent flooded forests derived from ALOS PALSAR's L-band SAR imagery from the dry and rainy season in Gabon.

The mapped flooded forests covered an area of 3,735 km^2^ (1,218 km^2^ and 2,517 km^2^ for permanent and flooded forests, respectively), representing 1.4% of the total area of Gabon mapped in our study (265,855 km^2^). Flooded forests in the north and north-east provinces are more distinctively separated from each other, showing relatively large patches of seasonally flooded forests in the north east (Fig 5A) and permanent flooded forests in the north. On the other hand, forests in the south and south-west provinces showed a mixed pattern of permanent and seasonally flooded.

### Qualitative validation and knowledge-based verification

**Fig 10:**
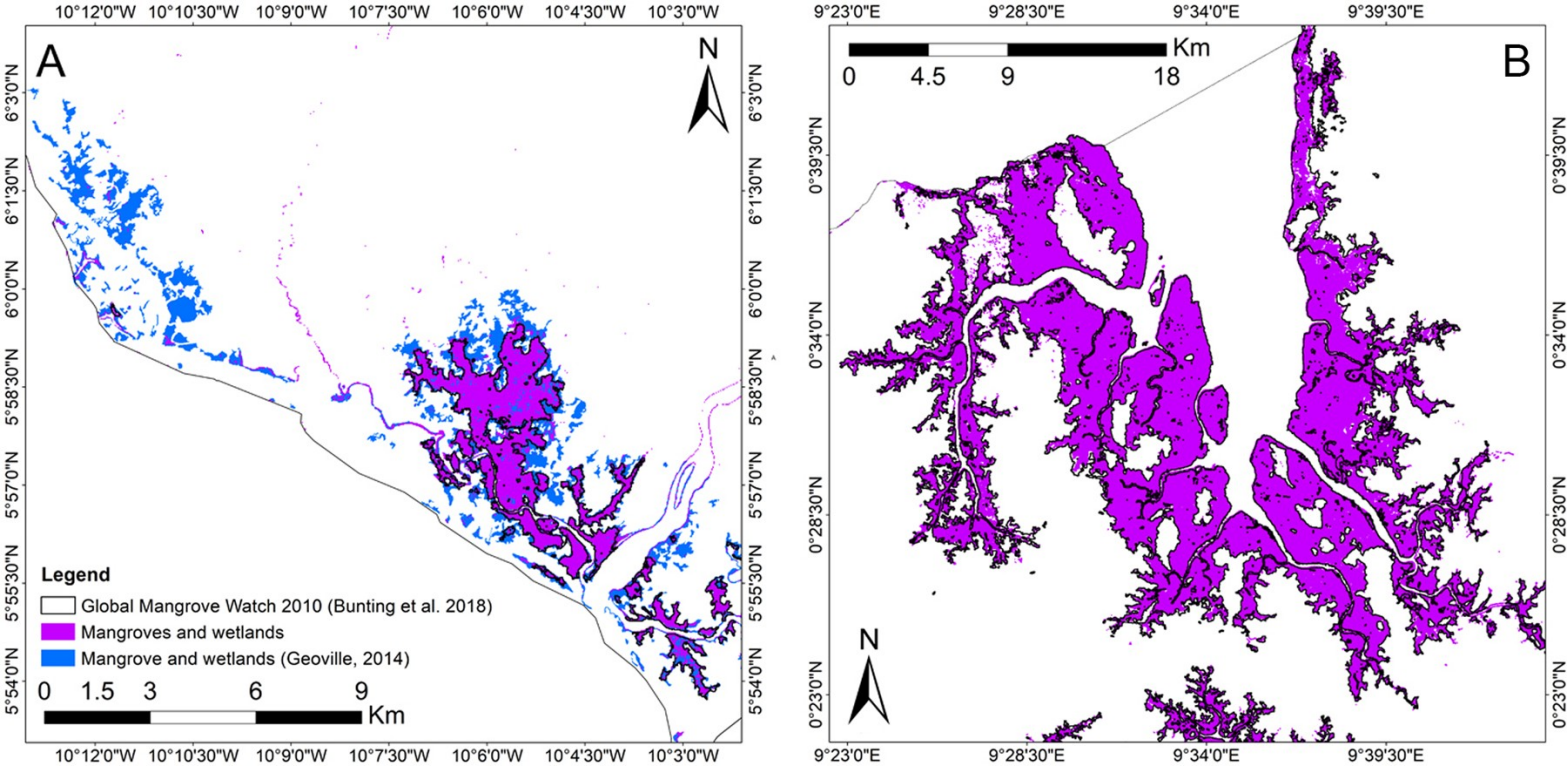
Comparison between our predicted land cover maps, the latest land cover land use product available for Liberia for 2014 and the global mangrove extent product for 2010. Comparisons were made between selected areas of the Mangroves and Wetland class from both Liberia (A) and Gabon (B) maps. While our maps show good agreement with the global extent product, an overestimation of Mangrove and Wetland areas by the previous land cover product available for Liberia was found (shown in blue).

As expected, our maps outmatched global products with coarser spatial resolutions as they show different land cover definitions, differences in data sources, methods and spatial resolution [13–17]. Comparable resolution (e.g. 30-m Globland30) and higher resolution (e.g. ‘S2 prototype LC 20m map of Africa 2016’) products also showed lower overall agreement with our maps. As an example, Fig 10 shows a visual comparison of Mangroves and Wetlands class (highlighted in purple) from our predicted land cover map and the latest 10-m land cover land use product available for Liberia for 2014 [68]. Note that the global mangrove extent for 2010 [31] was also added for comparison. Our map yielded a total area of 223.51 Km^2^ of mangroves in Liberia, while the global mangrove extent product for 2010 reported a mangrove extent of 191.6 Km^2^. The previous land cover product for Liberia showed an area of Mangroves of 371.58 Km^2^, roughly twice as large. A qualitative visual assessment of this product showed that large areas of dense tree covered areas was misclassified as Mangroves (Fig 10A). For Gabon, our land cover map yielded an area of 1,626.8 Km^2^ for Mangroves while the global mangrove extent product showed an extent of 1768.1 km^2^. Thus, our maps showed very good overall agreement with the global mangrove extent product for 2010 for both Liberia and Gabon. The pre-masking phase combined with the random forest model proved to be able to successfully distinguish mangrove areas from other vegetation classes. Therefore, no significant overestimation or underestimation of mangrove extent was observed in our maps.

The verification of land cover data is an important process to identify inconsistency and to remove, or minimize, errors in the dataset. We performed a knowledge-based verification of our map with the help of the local community and scientists in a workshop in Monrovia, Liberia. Attendees were part of the Environmental Protection Agency (EPA), Conservation International Liberia (CI–Liberia), Forestry Development Authority (FDA), Ministry of Agriculture, Ministry of Finance and Developing Planning, Liberia Institute of Statistics and Geo-Information Systems (LISGIS), National Fisheries and Aquaculture Authority, University of Liberia, Flora and Fauna International, Society for the Conservation of Nature of Liberia, among others (S4 Fig). The land cover map was presented in both printed and digital formats and the participants had the opportunity to visually inspect the product and provide feedback regarding its thematic content based on personal knowledge and location of key areas across the country. Feedback was received through direct interaction with the attendees as well as through written forms (S5 Fig). Generally, the attendees responded positively to the final map in terms of content and overall spatial distribution of land cover classes.

## Discussion

### Landsat composite

Using multiple years of Landsat data is the only way to attain a wall-to-wall high quality cloud-free composites for cloud-prone areas like Liberia and Gabon (S2 Fig). However, this approach may lead to contamination from older pixels. Depending on the number of years, the availability of observations at a given location and the aggregation function used (i.e. median), each pixel may belong to a specific year among those used in the image collection, or be its median value across those years. This contamination can introduce issues at several levels, mainly in locations or time periods with relatively fast land use change (i.e. annual crops). In our study, in Liberia, high temporal contamination was unlikely to be an issue as we focused on seasonal composites over three years instead of using the entire time period of observations. For Gabon, the entire time period of observation was used. This has also been shown in other studies (e.g. [44]) that have focused on three years composite to attain a cloud-free composite for the African continent, most likely due to the cloud prevalence over countries along the Gulf of Guinea.

### Classification

In general, water bodies exhibit a distinct spectral signature in Landsat TM/ETM+/OLI imagery compared to other land cover types. The Random Forest classification was relatively straight forward and based on the MNDWI. For the majority of mapped areas, the method worked well. Moreover, our water bodies mask had good general spatial agreements with the European Commission’s Joint Research Centre Global Surface Water mask [69].

In the case of Mangroves and Wetlands, the spectral diversity within this class is significant compared to water bodies. The successful extraction of Mangroves and wetlands in our map depended, primarily, on our masking phase and as well as the its spectral characteristics. A knowledge-based verification based on overall elevation (i.e. the likelihood of occurrence of Mangroves decreases as we move inland and the overall elevation increases) was proven useful to avoid confusion with other vegetation classes across Liberia and Gabon. A comparative assessment with the global mangrove extent for 2010 (Fig 10) showed that our mangrove and wetlands mask was in overall good agreement with the extent observed by the authors. In comparison with their global product, our maps showed an extent of 31.9 Km^2^ larger and 141.3 Km^2^ smaller for Liberia and Gabon, respectively.

Artificial surfaces (human settlements) in Liberia and Gabon consists of urban areas, roads, rural settlements, which are primarily based on asphalts and concrete (urban areas) and bare soil and other materials (rural settlements). Thus, being spectrally similar to bare areas. Both masking phase based on spectral information and human settlement locations was helpful to reduce confusion between these two classes. Once, human settlements were mapped, the classification of bare areas was fairly straightforward.

Cultivated land in Liberia and Gabon primarily consists of woody commodity crops (oil palm and rubber trees). The spectra of these woody crops are identical with those of natural vegetation. However, these areas generally had regular distribution patterns such as square or rectangles. The availability of forest concessions datasets for both countries was of paramount importance to constrain the classification of woody crop areas within the area they are likely to be found and avoid misclassification with other vegetation classes. In order to assess the overall quality of our product, we compared it with the latest land cover product available for Liberia [68]. A comparative visual assessment indicated that it does not include an industrial plantation class. Thus, areas of oil palm and rubber plantations are being classified mostly as grassland and accounted for an area of nearly 6,250 km^2^, thirteen times larger than the area we report here (453 km^2^).

After completing the classification of water bodies, mangroves and wetlands, artificial surfaces, barren land, ecosystem complex (for Liberia only) and woody crops, it became relatively straightforward to take the first step in classifying the tree covered areas classes, mixed vegetation and grassland classes because a large number of pixels with spectral similarity to these land cover types were masked out by the previous procedures. However, these classes are similar to each other, which may lead to confusion between classes. For instance, most nation-wide land cover products in Liberia and Gabon, along with global LCLU datasets (e.g [13–17]) tend to only show one forest (or tree covered area) class. Our goal was not to map too many classes where achieving high accuracies becomes complicated, but to include relevant classes from an ecosystem accounting and valuation perspective that are relatively simple for yearly replicating.

We found that the biggest difficulty in mapping different forest classes in Liberia and Gabon was: 1) complexity in defining them; 2) collecting sufficient ground truth training samples as these forests are usually in difficult access areas in northern Liberia and central and northern Gabon, and; 3) spectral similarity between tree covered areas in secondary growth and mixed vegetation in Liberia and flooded forests in Gabon. Apart from the class-specific challenges, we found that the lack of cloud-free observation is the main issue in working with classification of cloud-prone areas such as Liberia and Gabon. Combining data from two or more sensors into a single data set may be in order to overcome this challenge. For instance, Senintel-2 A/B combined with Landsat-8 would provide the ability to composite over a shorter period of time potentially enabling a change map for conservation over a shorter time interval. In fact, [70] have presented the Harmonized Landsat and Sentinel-2 (HLS) project, which aims to provide near-daily reflectance time series as though it came from a single sensor. However, currently the HLS v1.3 data set covers about 7.3% of the global terrestrial lands and which does not include Liberia or Gabon.

Although based on Landsat 8 OLI imagery only, the adopted binary classification strategy has shown the ability to help reduce ‘salt and pepper’ effects as only a reduced number of pixels were classified at each iteration. This strategy has also the advantage of mapped land cover classes being “independent” from each other, allowing revisit to a given land cover class if any further adjustment is needed without interfering on previously mapped classes. Even though our method shows clear advantages over a more traditional one-step classification approach, we acknowledge that the order in which the classification is performed may, to some extent, introduce commission and omission errors into the final output. However, it is unlikely that a different order will produce a highly different output and potentially compromise the overall accuracy of the final map. Moreover, the national experts’ feedback further assisted in refining the final classification output. We further acknowledge that our land cover map was intended to show the main land cover classes within Liberia to fit a broad range of applications. Specifically, we intend our maps to support the ongoing efforts for natural capital accounting and valuation of ecosystem services in Liberia and Gabon. Moreover, thanks to GEE’s computational capabilities and easy-to-implement RF classifier, our land cover map can be produced in a matter of a few hours. In fact, with our semi-automated methodology scientists and policy makers can generate similar maps aiming for specific needs or land cover classes not available in global land cover datasets or different areas and regions around the globe.

### GEE and other land cover products

Land cover datasets from GEE are often produced using the built-in Random Forest classifier (e.g. [25,27,29,38,44]). Coupled with GEE’s computing power and infrastructure, Random forest’s inherent capabilities of dealing with large amounts of training sample sets offered us the possibility for more and better representability of the different land cover classes and, in turn, better final product. On the other hand, readily available datasets that were produced outside of GEE’s cloud environment may present misclassification errors due to lack of computational power to deal with large training sample sets and less robust classification methods (e.g. maximum likelihood or decision trees based models).

Due to differences in data sources, land cover definitions, methods and spatial resolution, direct comparison of accuracy and thematic content between land cover products is challenging. However, qualitative comparisons between products with comparable resolutions can be made. As an example, we showed a visual comparison between our land cover map and the latest 10-m land cover land use product available for Liberia for 2014 on Fig 10. Although the latest Liberia land cover product has a higher spatial resolution, we attribute its lower overall thematic quality due to a less robust classification approach (hybrid unsupervised and supervised pixel based classification techniques such as maximum likelihood classifiers). Random forest-based land cover datasets will generally have an overall higher quality and accuracy than other land cover outputs from other classifiers [25–29]. In this study we showed that our integrated method of pixel-based classification using random forest classifier and ancillary data combined with pre-classification masking phases produced a superior final product, when compared to other RF-based land cover products produced within GEE’s cloud computing environment (e.g ‘S2 prototype LC 20m map of Africa 2016’).

While land cover classification and mapping is relatively well established, all the research that has been devoted to understanding the importance of the ecosystems services is challenging the remote sensing research community to refine what is already known about land cover classification and develop new methods and approaches. In this study, our approach aimed to capturing the ecosystem heterogeneity in Liberia and Gabon that will often present unique ecosystem services and are relevant to each nation’s natural capital accounting framework. From the natural capital accounting perspective, it is of paramount importance to have a thematically accurate land cover dataset from which the land cover and, consequently, ecosystem extent is derived. This ecosystem extend will be the baseline to quantify and evaluate the ecosystem services within the natural capital accounting framework. Therefore, we have to minimize the errors in land cover extent estimates that rise from land cover datasets derived from coarse resolution imagery and less robust classification methods.

## Conclusions

This study demonstrated an efficient, cost effective and reliable approach to produce a land cover map in a region of the world that has been difficult for optical remote sensing to produce reliable estimates of land cover classes for conservation due to the need for dense time-series to screen clouds and cloud shadows. Our study also aimed to fill the observed gap in up to date land cover data at national scale for Liberia and Gabon. The proposed classification approach along with the accuracy assessment results showed that the land cover maps for Liberia and Gabon had the level of accuracy which is within the ‘high accuracy’ range accepted by the remote sensing community. Our land cover maps produced an overall accuracy of 83% and 81% for Liberia and Gabon, respectively, for 10 land cover classes and outperformed the global land cover datasets compared in this study. The land cover map for Liberia had a knowledge based verification aspect based on local stakeholders’ feedback. Aside from a high overall accuracy, our methodology produced a map that was highly accepted amongst all the stakeholders and experts in Liberia. We conclude that our approach, while relatively simple and highly replicable, is robust and capable of producing land cover maps that are highly suitable for a broad range of applications, including natural capital accounting.

The land cover maps produced here are now being used as the basis for the development of an ecosystem extent classification which involves subdividing land cover classes (e.g., forest) into ecologically distinct ecosystem types using generalized dissimilarity modelling (Honzák et al., in prep). As part of the commitment of Liberia and Gabon to the GDSA, both countries need to account for the distinct stocks and flows of ecosystem services associated with each ecosystem type. The land cover map has already provided the foundation for an ecosystem classification that has identified areas providing essential natural capital along Liberia’s coast (especially priority mangrove sites) as well as to guide the development of a sustainable landscape connecting the inland mountains to the ocean. These results are helping the government of Liberia to prioritize funding, develop management strategies and guide spatial planning at the national scale. Since natural capital accounting requires tracking trends over time, the same approach to map land cover described here will provide a standardized and cost-effective approach to periodically update maps of ecosystem extent and condition that form the foundation for a national accounting framework that ensures inclusion of nature’s benefits into decision-making. Moreover, we aim to produce a baseline up to date land cover map for different countries committed to the Gaborone Declaration for Sustainability in Africa to support their efforts on integrating the value of nature into their national policies.

## Supporting information

S1 Fig **2015 annual mosaic composites of Landsat 8 (RGB 432) (A and B) and Sentinel-2 (RGB 321) (C and D) for Liberia and Gabon showing areas with no data after cloud masking.** Multi-year mosaics are necessary to attain a complete cloud-free mosaic composite for both countries.
(TIF)

S2 Fig Landsat scene availability on GEE from 1984 to 2017 (top) and overall percentage of cloud cover for these landsat scenes over Liberia and Gabon. Liberia and Gabon are covered by 11 and 21 Landsat WRS-2 grids, respectively. During the lifetime of the Landsat Missions 4–8 more than 2440 images were collected for Liberia and 3169 images were collected over Gabon. Both countries show very high number of scenes with more than 50% of its area covered by clouds.
(TIF)

S3 Fig Example of photographs taken on four cardinal points in areas of Grassland (top row), woody crops (middle row) and tree covered areas (dense/primary) (bottom row). (TIF)

S4 Fig Participant list for the expert workshop in Liberia. (JPG)

S5 Fig Example of written form in which workshop attendees provided their feedback regarding overall accuracy and thematic content for the Liberia land cover map. (TIF)
