# Supplementary figures and images for: Cloud-computing and machine learning in support of country-level land cover and ecosystem extent mapping in Liberia and Gabon
Source: PLoS One. 2020 Jan 10;15(1):e0227438. doi: 10.1371/journal.pone.0227438 (PMC6953846; doi:10.1371/journal.pone.0227438)

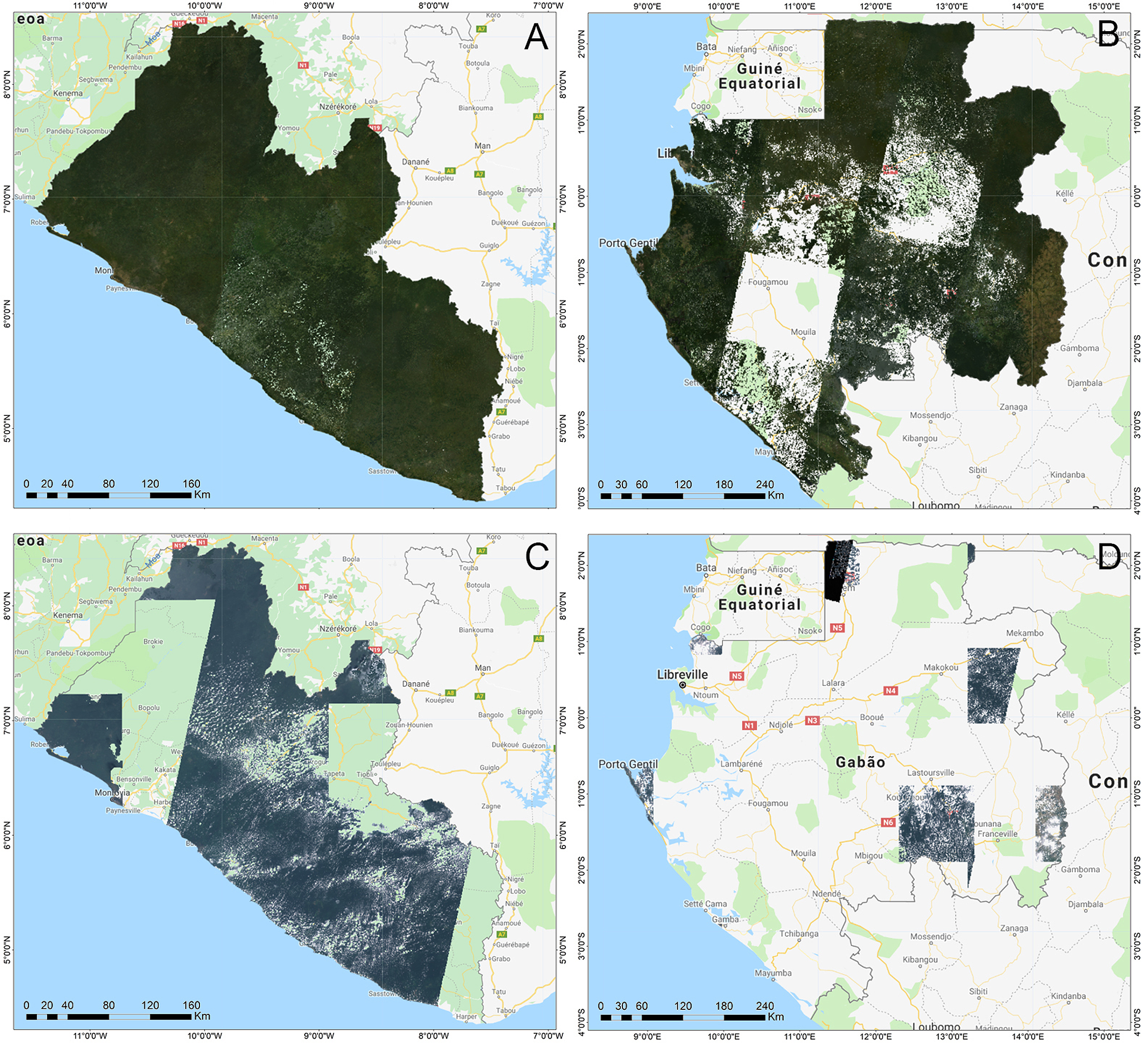

Supplement: S1 Fig — 2015 annual mosaic composites of Landsat 8 (RGB 432) (A and B) and Sentinel-2 (RGB 321) (C and D) for Liberia and Gabon showing areas with no data after cloud masking. Multi-year mosaics are necessary to attain a complete cloud-free mosaic composite for both countries. (TIF) [file pone.0227438.s001.tif]

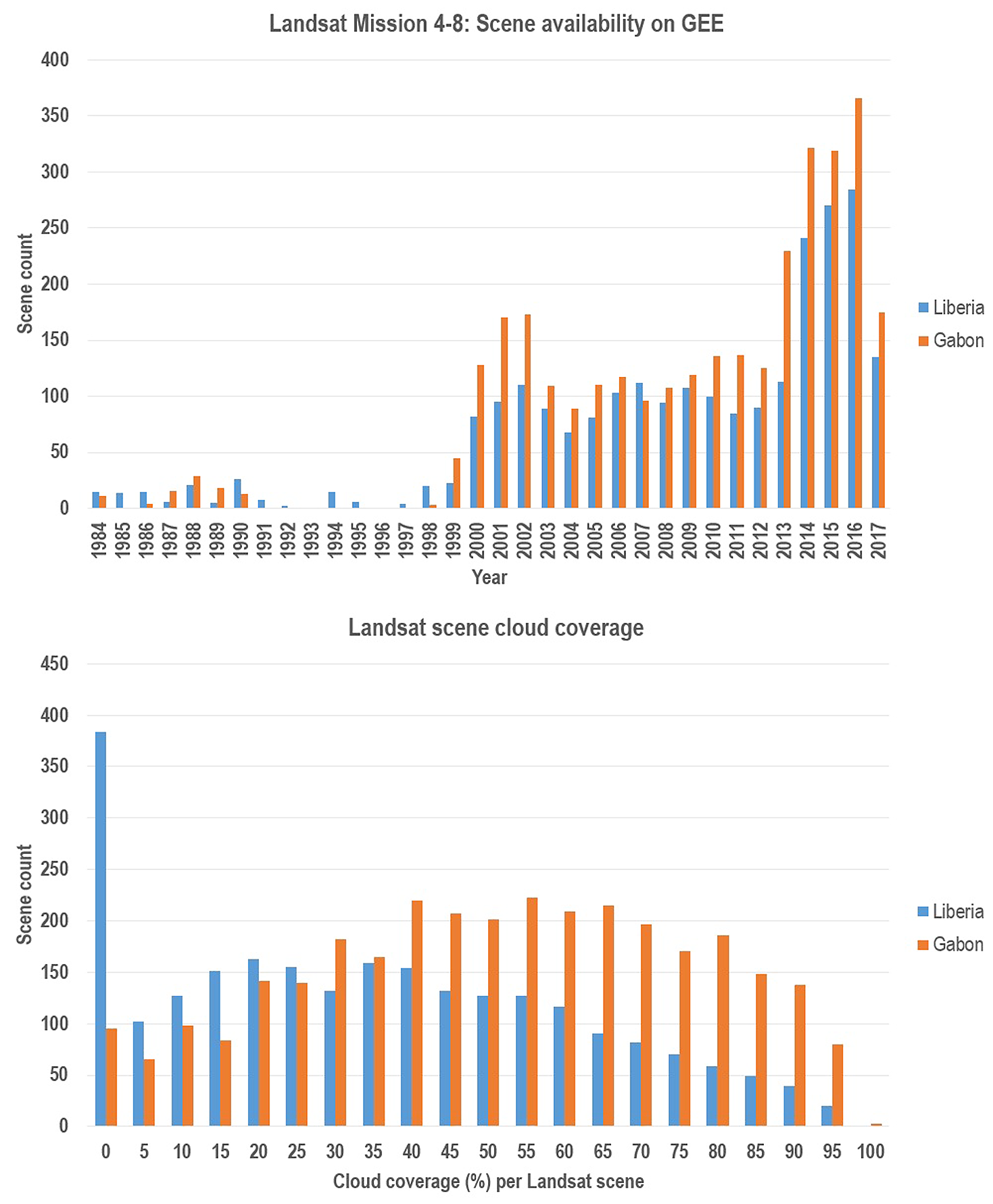

Supplement: S2 Fig — Liberia and Gabon are covered by 11 and 21 Landsat WRS-2 grids, respectively. During the lifetime of the Landsat Missions 4–8 more than 2440 images were collected for Liberia and 3169 images were collected over Gabon. Both countries show very high number of scenes with more than 50% of its area covered by clouds. (TIF) [file pone.0227438.s002.tif]

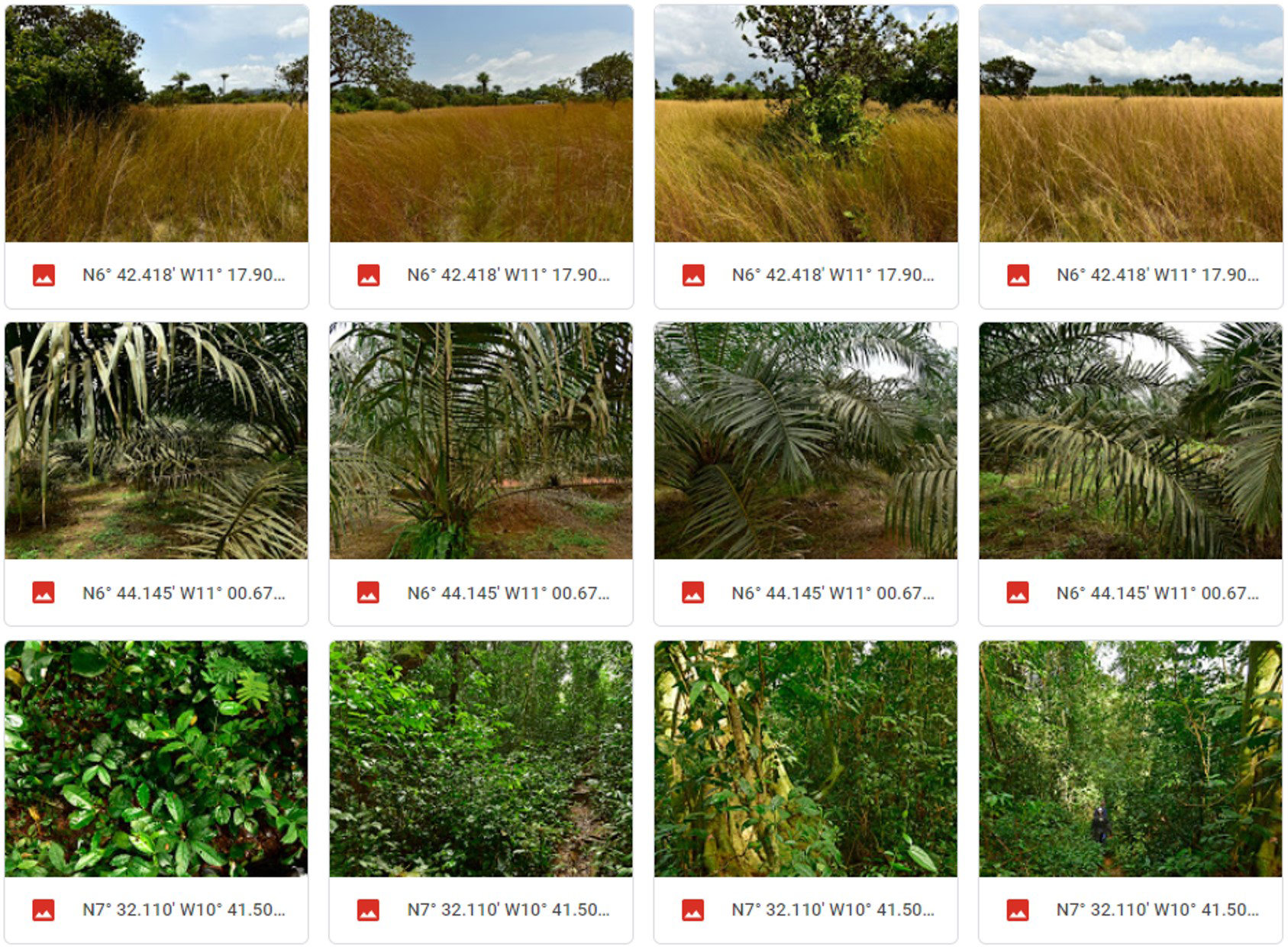

Supplement: S3 Fig — (TIF) [file pone.0227438.s003.tif]

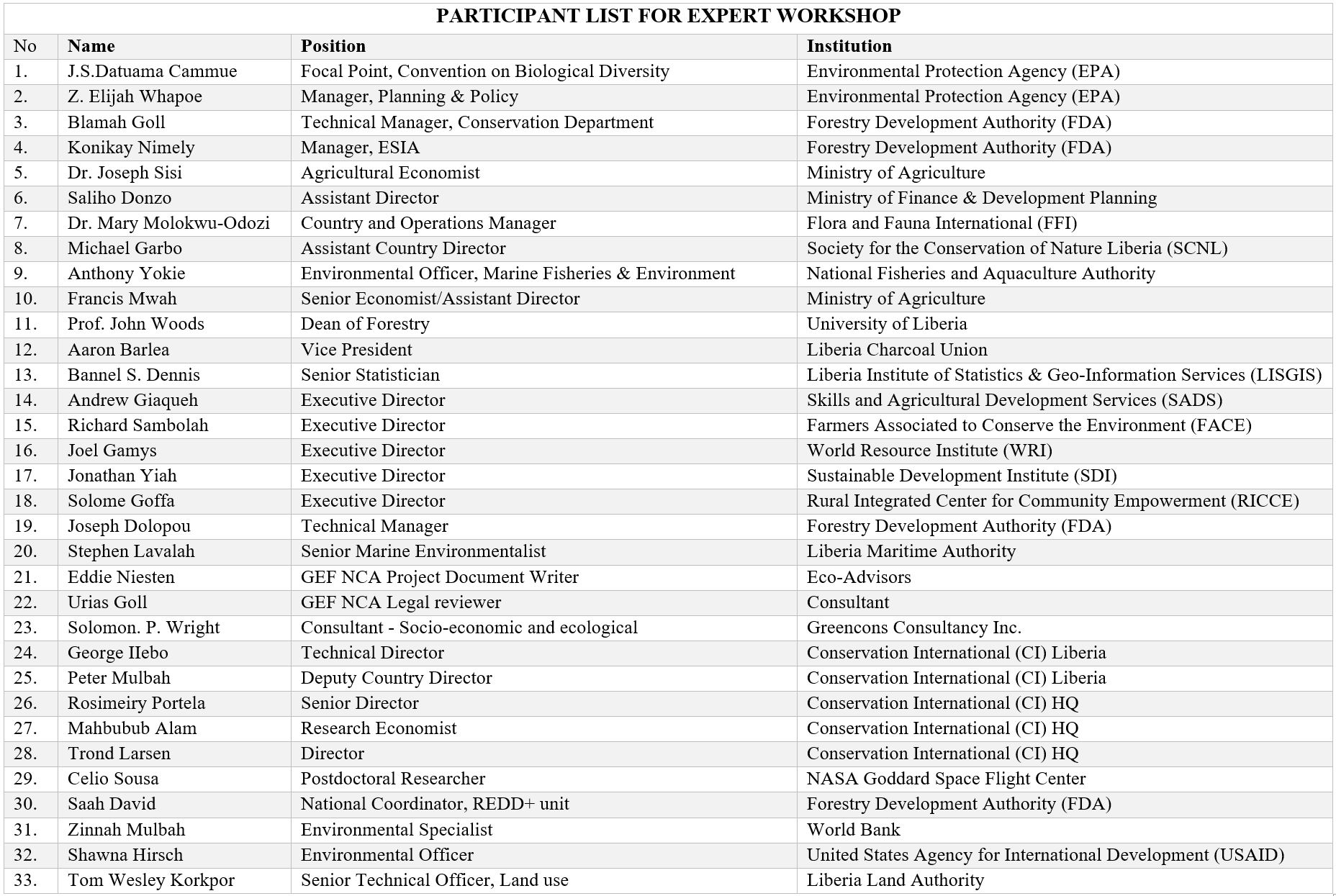

Supplement: S4 Fig — (JPG) [file pone.0227438.s004.JPG]

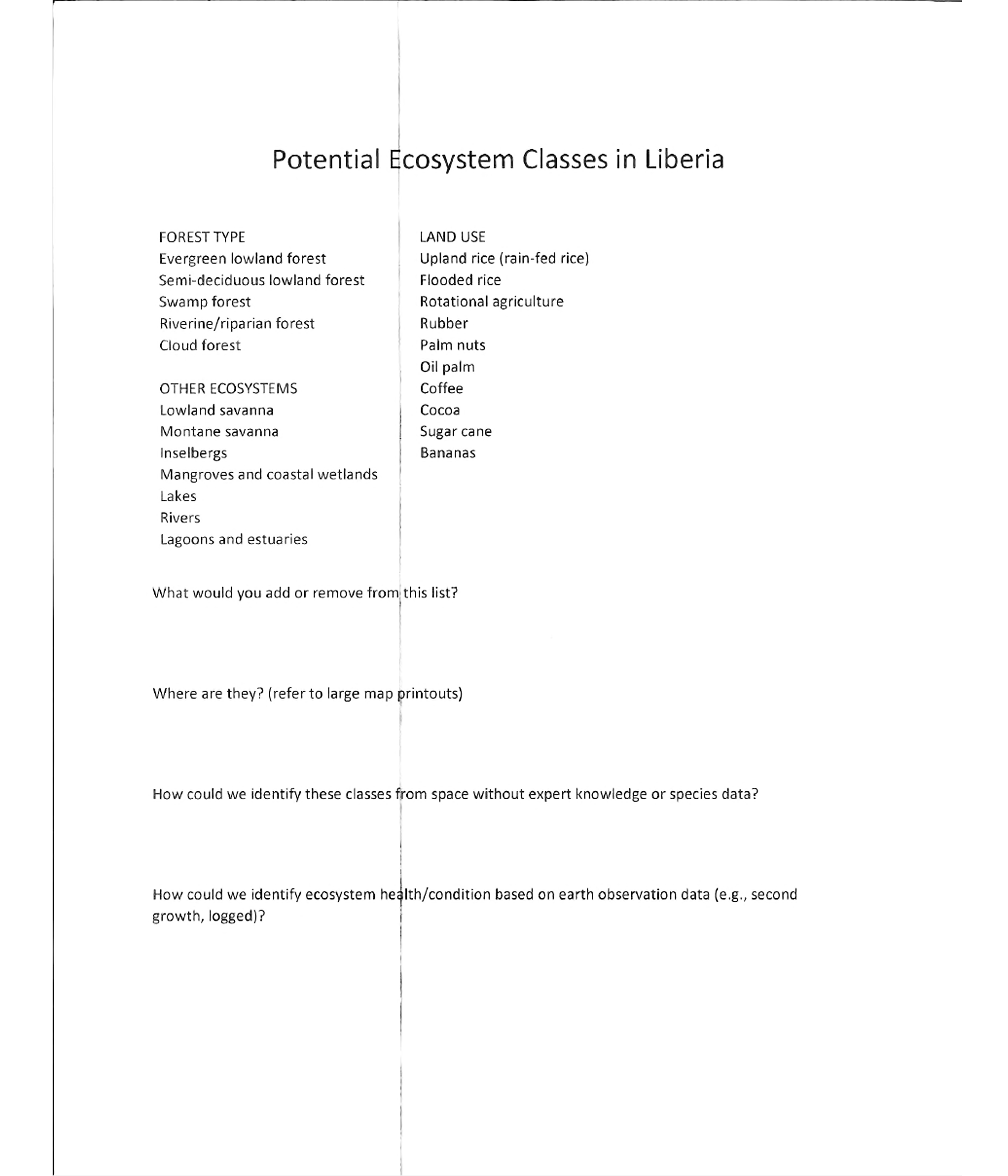

Supplement: S5 Fig — (TIF) [file pone.0227438.s005.tif]
